# Assessing perception of mattering in a cross-cultural university context: validity and reliability of the Italian and Hungarian versions of the University Mattering Scale (UM-S)

**DOI:** 10.3389/fpsyg.2024.1502661

**Published:** 2024-12-02

**Authors:** Camilla Matera, Zsuzsanna Katalin Papp, Monica Paradisi, Chiara Pieri, Jonathan Catling, Amanda Nerini

**Affiliations:** ^1^Laboratory of Social Psychology, Department of Education, Languages, Intercultures, Literatures and Psychology, University of Florence, Florence, Italy; ^2^Institute of Mental Health, Semmelweis University, Budapest, Hungary; ^3^School of Psychology, University of Birmingham, Birmingham, United Kingdom

**Keywords:** university students, mattering, well-being, academic success, validation, Italy, Hungary

## Abstract

The present study aimed to gather evidence on the validity and reliability of the Italian and Hungarian versions of the University Mattering Scale (UM-S). This 10-item scale assesses university students’ perceptions of mattering across three dimensions: Awareness, Importance, and Reliance. University students from Italy (*n* = 210) and Hungary (*n* = 191) completed a questionnaire that included the adapted UM-S, along with measures of societal mattering, social support, well-being, and academic self-efficacy. A confirmatory factor analysis (CFA) supported the three-factor structure of the scale in both contexts. The scale demonstrated high internal consistency, providing evidence of its reliability. Consistent with the original version, both the Italian and Hungarian UM-S showed good discriminant and convergent validity, as evidenced by its association with instruments measuring perceived social support and societal mattering. Additionally, the scale showed strong criterion-related and incremental validity; university mattering significantly predicted students’ well-being and academic self-efficacy, even after controlling for perceived social support. Furthermore, the scale was partially invariant across countries at the scalar level. A comparison of UM-S scores between the two groups revealed that Hungarian students perceived higher levels of university mattering than their Italian counterparts. In conclusion, the Italian and Hungarian versions of the UM-S are appropriate for use in academic contexts to assess students’ sense of being valued by their university. The instrument, having been shown to be both valid and reliable, is suitable for both research and intervention purposes.

## 1 Introduction

The university experience, filled with both opportunities and challenges, is a pivotal time in many young people’s lives (Brett et al., 2023). While the academic environment often promotes growth and independence, some students may find it overwhelming due to pressures related to academics, forming new social connections, and financial difficulties (Brett et al., 2023). These challenges can negatively impact students’ well-being, increasing the risk of developing mental health issues (Auerbach et al., 2018). A survey of 13,984 first-year students from 19 colleges across eight countries revealed a high unmet need for mental health treatment, alongside significant rates of suicidal thoughts and behaviors (Bruffaerts et al., 2019). Roughly a third of students reported symptoms of a mental health disorder, with major depressive episodes (21.2%) and generalized anxiety disorder (18.6%) being the most prevalent (Bruffaerts et al., 2019).

The perception of mattering has been identified as a key factor in fostering students’ well-being (Flett et al., 2019). Mattering can be defined as the personal sense of being important, of making a difference in others’ lives, and being valued (Rosenberg and McCullough, 1981). According to Rosenberg and McCullough (1981), it has three main components: the perception that others pay attention to us, the belief that others consider us important, and the sense that others depend on us. Similarly, Elliott et al. (2004) identified three dimensions: awareness, which is the sense that others notice us and are attentive to our experiences and emotions; importance, which refers to the belief that those around us view us as significant; and reliance, the extent to which others depend on us.

Mattering is a fundamental element in shaping one’s self-concept (Marshall, 2004) and is strongly associated with various aspects of health and well-being (Paradisi et al., 2024). This is particularly true for university students (Flett et al., 2019), for whom feeling recognized and supported by their institution is essential to their sense of belonging (Schlossberg, 1989). Students who do not feel valued by their institution are more likely to experience depressive symptoms (Dixon and Robinson Karpius, 2008; Flett et al., 2012), social anxiety (Flett et al., 2016), and, in extreme cases, suicidal behavior (Flett et al., 2019). A lack of mattering is linked to increased academic stress (Dixon Rayle and Chung, 2007), which can impair performance (Sand et al., 2004) and, in severe cases, lead to university dropout (Dixon Rayle and Chung, 2007; Lovitts, 2001) and mental health issues (Misra et al., 2000). On a more positive note, mattering can enhance academic success and help students adjust to the university environment (Flett et al., 2022; France, 2011). Students who feel appreciated by their university tend to have higher self-esteem and are more goal-oriented (France and Finney, 2010).

Several scales have been developed to assess university students’ perception of mattering. The first was the Mattering Scale for Adult Students in Higher Education (Schlossberg et al., 1990), which examined mattering in relation to both peers and university administration. However, some critics argue that this scale was not based on Rosenberg and McCullough’s (1981) theory of mattering, raising concerns about its construct validity (France, 2011). Tovar et al. (2009) developed the College Mattering Inventory, a 29-item measure with six subscales, each addressing different aspects of university mattering. However, the scale has been critiqued for the low validity of some items and for overlapping with the concept of belonging (Flett et al., 2019).

In response to the limitations of previous instruments, France and Finney developed the University Mattering Scale. They adapted the Mattering Index (Elliott et al., 2004) for the academic context, resulting in a 24-item scale with three subscales aligned with Rosenberg and McCullough’s (1981) components of mattering. Given the robust conceptual framework and strong validity and reliability of the scale, several versions have been developed: the Revised University Mattering Scale (France, 2011), the Unified University Mattering Scale (France, 2011), and the Unified Measure of University Mattering–Short Form (UM-S; Moschella and Banyard, 2021). The latter was adapted from the UMS (France and Finney, 2010) to reduce survey fatigue and improve focus during completion, as suggested by Porter et al. (2004). This 10-item measure takes about 5 min to complete. Exploratory factor analysis on the UM-S confirmed a three-factor structure, consistent with the original scale. The first factor consists of four items representing the awareness subscale, the second factor includes four items addressing the importance dimension, and the third factor is composed of two items related to reliance. Confirmatory factor analysis (CFA) further supported this three-factor structure. Reliability, measured by Cronbach’s alpha, was excellent across all subscales (Awareness = 0.90, Importance = 0.94, and Reliance = 0.89). The scale presented good convergent and divergent validity. As regards proof of concept, students who reported higher levels of university mattering also experienced greater well-being and showed increased persistence in their studies. The authors confirmed that the shortened measure retained the psychometric properties of the full version (Moschella and Banyard, 2021).

## 2 The present study

University students’ well-being is gaining increasing attention in many countries, highlighting the importance of having reliable instruments to assess this construct across different sociocultural contexts. The present study aims to test the validity and reliability of the Italian and Hungarian adaptations of the University Mattering Scale (UM-S). Reports suggest that Italian and Hungarian students face similar challenges in terms of mental health and well-being (National Youth Council Study Center, 2023). According to a report by the National Youth Council Study Center (2023), 30% of university students in Italy experience poor mental health, with an additional 18% reporting suboptimal physical health. Many students (40%) indicate that their mental health negatively affects their daily activities and relationships. The primary sources of academic stress include strained relationships with professors (50.4%), difficulties in making friends in class (34.2%), academic competition (24.3%), parental expectations (18.9%), and concerns about academic performance related to future job opportunities (13.5%). Similarly, a 2021 survey of over 7,600 Hungarian university students found that 40% reported experiencing low or moderate mood disorders (Karner et al., 2021). Interventions to improve students’ mental health and well-being are needed in both sociocultural contexts. Since there are no validated measures for assessing university mattering in either Italy or Hungary, we aimed to test the psychometric properties of the UM-S in these two contexts.

We first translated the UM-S from English into Italian and Hungarian. We then tested the scale’s three-factor structure through CFA and assessed its reliability in terms of internal consistency. To evaluate the scale’s convergent validity, we examined its correlation with a measure of societal mattering. Evidence for discriminant validity was obtained by analyzing the associations between UM-S scores and a measure of perceived social support, which is related but distinct from university mattering. We tested the criterion-related validity of the UM-S by analyzing its associations with well-being and academic self-efficacy. To assess the scale’s incremental validity, we performed multiple linear regressions, with UM-S as a predictor of each key outcome, controlling for perceived social support. Finally, we tested the measurement invariance of the UM-S across the two countries and conducted a comparison of university mattering perceptions between Italian and Hungarian students.

## 3 Materials and methods

### 3.1 Procedure

Some students completed the questionnaire in person in small groups at the end of some university lessons. Other students, recruited online using a snowball sampling method via social media platforms (e.g., Facebook, Instagram), completed it online. Students were invited to take part in a survey on their university experience and wellbeing. Participation was voluntary basis and no incentives were offered. Before compiling the questionnaire, all participants had to provide informed consent. The study, funded by EUniWell – European University for Wellbeing, was approved by the Ethics Committee of the University of Florence (n. 0030489/08-02-2024) and by Semmelweis University Regional and Institutional Committee of Science and Research Ethics (SE RKEB 259/2023). All the data were collected between February 2024 and June 2024.

### 3.2 Participants

In Italy participants were 201 university students (mean age = 22.04, SD = 1.91); 58% identified themselves as women, 42% as men, and 0.1% choosing another gender identity. Most participants (93.6%) were attending a Bachelor’s degree program at the University of Florence, while a small part of the sample consisted of students from the University of Bologna (6.4%).

In Hungary participants were 192 university students (mean age = 24.87, SD = 7.14). 75.9% identified as women, 20.9% as men, 1.6% defined themselves differently and 1.6% did not want to respond. All participants were active students from Semmelweis University. Most of them (84.1%) were studying in their first 3 years.

### 3.3 Measures

Participants were administered a questionnaire containing the following scales.

#### 3.3.1 University Mattering Scale - short form

Developed by Moschella and Banyard (UM-S, 2021), this 10-item scale evaluates perception of mattering in the academic environment. The Italian and the Hungarian versions were obtained following broadly accepted translation guidelines (International Test Commission, 2017). First of all, the whole research team, composed of scholars who are both experts of the assessed construct and are familiar with the cultural groups being tested (respectively Italian and Hungarian university students), evaluated the legimitacy of assessing university mattering in these two cultural/linguistic groups (International Test Commission, 2017). In both countries two highly qualified translators were recruited adopting a forward and backward translation design. All the items were translated into Italian/Hungarian by a native Italian/Hungarian speaker who lived in the target locale and had good knowledge of assessment principles. The Italian/Hungarian research team carefully revised this translation and judged it as adequate. Consequently, another native Italian/Hungarian speaker, who was proficient in English and was not affiliated with the study, back translated this Italian/Hungarian version into English. The back-translated English version and the original one were carefully compared by the Italian/Hungarian research team, to arrive at a final Italian/Hungarian version. No relevant discrepancies emerged and both the Italiand and Hungarian adaptations were considered adequate for being adiministered to university students in the two countries. The scale comprises 3 subscales: Awareness (e.g., “The majority of people at my university recognize me”), Importance (e.g., “People in my university community do not care about my personal welfare.”), and Reliance (e.g., “When people at my university need help, they come to me.”). Responses are provided on a Likert scale ranging from 1 (“Strongly disagree”) to 6 (“Strongly agree”). When administered to students, no issues concerning the scale’s instructions, content or format emerged in either Italy or Hungary.

#### 3.3.2 Societal Mattering Scale

The Societal Mattering Scale (Schmidt, 2018; Italian version by Paradisi et al., 2023) measures individuals’ perception of being important to the broader society. It comprises 9 items (e.g., “The people in the society value me as a person”) with responses on a Likert scale (1 = “Strongly Disagree”; 5 = “Strongly Agree”). Since this scale was not yet adapted to the Hungarian context, we tested its factorial structure through CFA and its reliability in terms of internal consistency using both the alpha (*α*) and the omega (*ω*) coefficients. CFA confirmed the unidimensional model of the Hungarian version of the Societal Mattering Scale with a good fit to the data after freeing error covariances between Items 2 and 3, items 9 and 4, and items 9 and 7 (*χ*^2^ = 54.705 *p* < 0.001, *χ*^2^/df = 2.28, RMSEA = 0.09, SRMR = 0.03, CFI = 0.97). Also reliability was very good (ITA *α* = 0.91, *ω* = 0.90; HUN *α* = 0.94, *ω* =0.94).

#### 3.3.3 Multidimensional scale of perceived social support

This 12-item scale (Zimet et al., 1988; Italian version by Prezza and Principato, 2002; Hungarian version by Papp-Zipernovszky et al., 2017) assesses perceived social support coming from different sources, corresponding to three subscales: Family (e.g., “My family really tries to help me”); Friends (e.g., “I can count on my friends when things go wrong”); Significant Other (e.g., “There is a special person who is around when I am in need”). Responses were given on a Likert scale ranging from 1 (“Very strongly disagree”) to 7 (“Very strongly agree”). Reliability was good (ITA *α* = 0.93, *ω* =0.92; HUN *α* = 0.86, *ω* =0.85).

#### 3.3.4 PERMA-Profiler

The PERMA-Profiler (Butler and Kern, 2016; Italian validation by Giangrasso, 2021; Hungarian validation by Varga et al., 2022) integrates the hedonic and eudaimonic approach to well-being (Seligman, 2011). It contains 15 items comprising 5 scales: Positive Emotions (e.g., “In general, how often do you feel joyful?”), Engagement (e.g., “How often do you become absorbed in what you are doing?”), Relationships (e.g., “To what extent do you receive help and support from others when you need it?”), Meaning (e.g., “In general, to what extent do you lead a purposeful and meaningful life?”), and Accomplishments (e.g., “How much of the time do you feel you are making progress toward accomplishing your goals?”). Responses were given on a Likert scale (1 = “Never”; 10 = “Always”). Reliability was very good (ITA *α* = 0.95, *ω* =0.95; HUN *α* = 0.93, *ω* =0.93).

#### 3.3.5 General Self Efficacy Scale applied to the academic context

To measure academic self-efficacy, we used the General Self Efficacy Scale (Schwarzer and Jerusalem, 1995; Italian version by Sibilia et al., 1995; Hungarian version by Kopp et al., 1995). To apply the scale to the academic context, participants were asked to give their answer referring to their university experience. The scale consists of 10 items (e.g., “I can always manage to solve difficult problems if I try hard enough”) with responses provided on a Likert scale from 1 (“Not true at all”) to 4 (“Exactly true”). Reliability was good (ITA *α* = 0.86, *ω* =0.86; HUN *α* = 0.82, *ω* =0.88).

### 3.4 Data analysis

CFA was implemented through AMOS 24 (Arbuckle, 2019). The fit of the model was examined using χ^2^/df, the Root Mean Square Error of Approximation (RMSEA), the Standardised Root Mean Square Residual (SRMR), the Comparative Fit Index (CFI), the Normed Fit Index (NFI) and the Incremental Fit Index (IFI). A *χ*^2^/df ratio not greater than 5 was deemed as acceptable (Schumacker and Lomax, 2004). The model fit was considered acceptable if CFI, NFI and IFI were higher than 0.90, RMSEA was between 0.08 and 0.10, and SRMR was lower than 0.08; a good fit was detected by CFI, NFI and IFI higher than 0.95, RMSEA lower than 0.08, and SRMR lower than 0.05 (Hooper et al., 2008). Cronbach’s alpha and McDonald’s omega were used to estimate internal consistency. Convergent (societal mattering), divergent (perceived social support) and criterion-related (well-being and academic self-efficacy) validity were examined through Pearson correlation coefficient; we conducted multiple regression analyses positing wellbeing and academic self-efficacy, respectively, as the criterion variables to test for incremental validity. Measurement invariance was assessed at the configural, metric and scalar levels (Vandenberg and Lance, 2000). Following the recommendations of Cheung and Rensvold (2002) and Chen (2007), as cut-off a combination of ΔCFI ≤0.010 and ΔRMSEA ≤0.015 was used. Differences between the two countries were examined through t test for independent samples.

## 4 Results

For all the items, the skew was lower than 2 and the kurtosis was lower than 7 (West et al., 1995). According to the established criteria, all the items were normally distributed (Supplementary Table S1).

**Figure 1:**
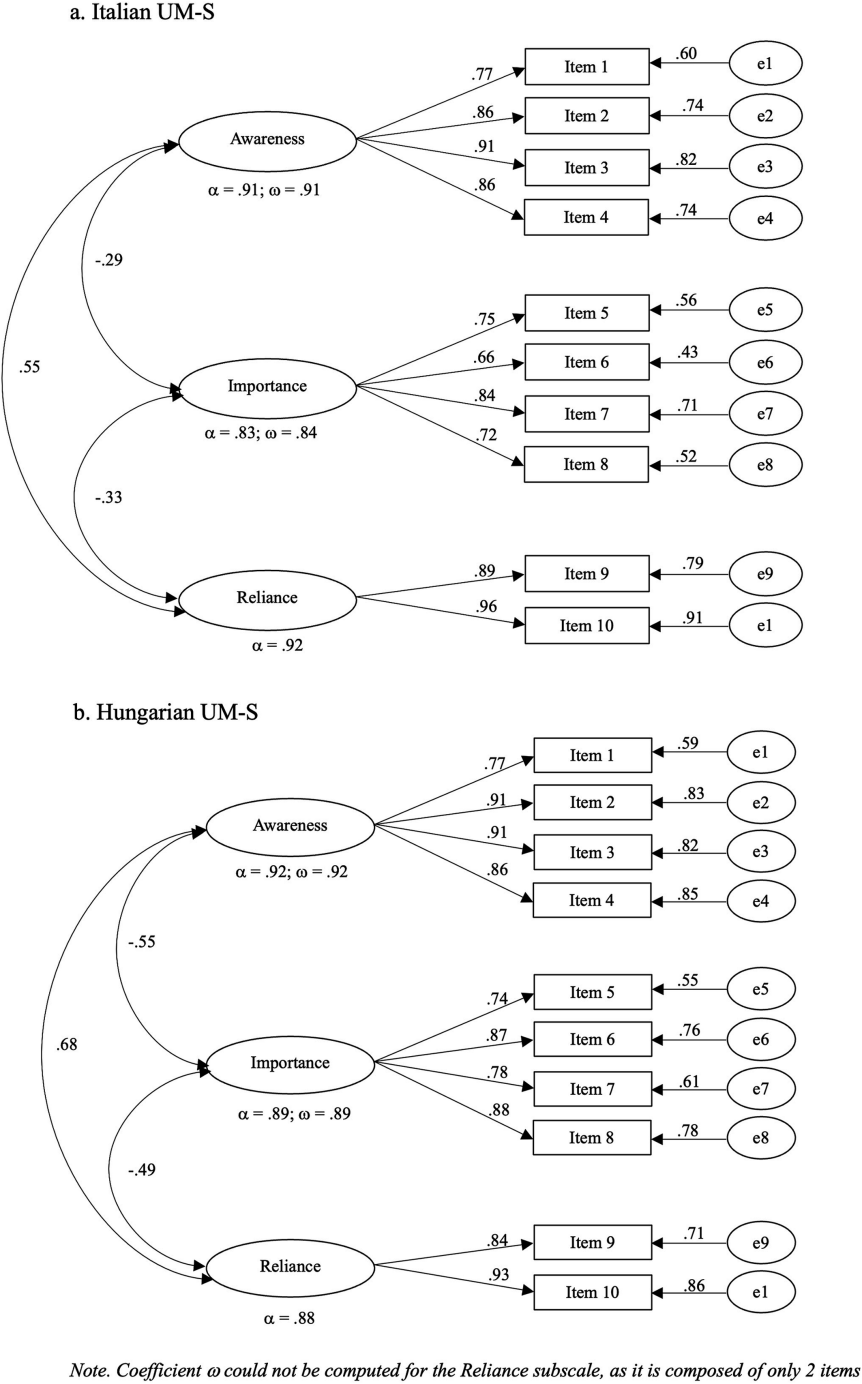
CFA of the Italian and Hungarian versions of the UM-S. **(A)** Italian UM-S; **(B)** Hungarian UM-S. Coefficient *ω* could not be computed for the reliance subscale, as it is composed of only 2 items.

The hypothesized three-factor model presented an excellent fit to the data for both Italian (*χ*^2^/df = 1.54, CFI = 0.99, NFI = 0.97, IFI = 0.99, RMSEA = 0.05 [0.018–0.075], SRMR = 0.03) and Hungarian participants (*χ*^2^/df = 2.23, CFI = 0.97, NFI = 0.95, IFI = 0.97, RMSEA = 0.08 [0.055–0.105], SRMR = 0.04). For both versions of the UM-S all factor loadings were greater than 0.65 (see Figure 1).

The internal consistency of both the Italian (*α* = 0.86; *ω* = 0.83) and Hungarian (*α* = 0.91; *ω* = 0.90) scales were very good, as well as the reliability of the three subscales (see Figure 1).

Table 1 summarizes the correlations between the UM-S and concurrent, discriminant and criterion-related measures.

**Table 1:** Descriptives statistics and correlations between the UM-S and measures of societal mattering, perceived social support, well-being and academic self-efficacy.

Correlations	Mean (SD)		1.	2.	3.	4.	5.
	Italy	Hungary					
1. University mattering	3.35 (0.88)	3.76 (1.16)	–	0.47	0.23	0.24	0.25
2. Societal mattering	2.64 (0.75)	2.74 (1.06)	0.56	–	0.27	0.27	0.39
3. Perceived social support	5.66 (1.21)	4.23 (0.68)	0.44	0.33	–	0.55	0.28
4. Well-being	6.55 (1.77)	6.86 (1.64)	0.53	0.63	0.56	–	0.46
5. Academic self-efficacy	2.81 (0.47)	2.89 (0.54)	0.41	0.53	0.30	0.51	–

Correlations results from the Italian sample are described above the diagonal; while results from the Hungarian sample are reported below the diagonal. All correlation are significant at *p* < 0.001.

Among Italian participants the UM-S showed a significant but small correlation with perceived social support, while the association with societal mattering was medium. These results provide evidence for the discriminant and concurrent validity of the UM-S, which is a measure of societal mattering in a specific context, but not a measure of perceived social support. Similarly, among Hungarian participants, the UM-S showed a significant medium correlation with social support and a large association with societal mattering.

To test the incremental validity of the scale, we conducted multiple regression analyses positing well-being and academic self-efficacy, respectively, as the criterion variable. At step 1 perceived social support was included, in order to control for its effect. At step 2 university mattering was entered. Both models were significant for Italian and Hungarian students (see Table 2). Perceived social support was a significant predictor of both well-being and academic self-efficacy. At step 2, university mattering emerged as a significant predictor of both well-being and academic self-efficacy, even after controlling for the effect of perceived social support.

**Table 2:** Hierarchical linear regression models.

		Regression model -well-being				Regression model - academic self-efficacy			
		*β*	*t*	*R* ^2^	Δ*R*^2^	*β*	*t*	*R* ^2^	Δ*R*^2^
Italy									
Step 1									
	Social support	0.55	9.32***	0.30***		0.28	4.10***	0.07***	
Step 2									
	Social support	0.52	8.68***	0.32***	0.02*	0.23	3.41***	0.10***	0.03**
	University mattering	0.12	1.93*			0.19	2.81**		
Hungary									
Step 1									
	Social support	0.56	8.97***	0.31***		0.30	4.22***	0.09***	
Step 2									
	Social support	0.40	6.23***	0.41***	0.10***	0.15	2.01*	0.18***	0.09***
	University mattering	0.36	5.61***			0.34	4.54***		

*p < 0.05; **p < 0.01; ***p < 0.001.

Finally, the UM-S resulted to be invariant at a metric level. Since it did not respect the cut-offs for the scalar level we tested for partial invariance, unconstraining the intercepts for items 6, 7 and 9. Even though ΔCFI was slightly over the recommended cut-off (see Table 3), according to Chen (2007) and Cheung and Rensvold (2002), the complexity of the model tested, namely the presence of three factors, can have affected the CFI index, so the measure can be considered nevertheless partial invariant at a scalar level.

**Table 3:** Measurement invariance across countries.

Model	*χ* ^2^_(gdl)_	*χ*^2^/gdl	CFI	RMSEA (IC 90%)	Model comparison	Δ*χ*^2^	ΔCFI	ΔRMSEA
Configural	142.52 (64)	2.23	0.970	0.056 (0.044–0.068)				
Metric	167.19 (71)	2.35	0.963	0.059 (0.047–0.070)	Metric vs. Configural	24.66 (7) *p* = 0.001	−0.007	0.003
Scalar	234.73 (81)	2.90	0.941	0.070 (0.059–0.080)	Scalar vs. Metric	67.54 (10) *p* = 0.000	−0.022	0.011
Partial scalar (item 6,7 and 9)	209.51 (78)	2.69	0.950	0.066 (0.055–0.076)	Partial Scalar vs. Metric	42.32 (7) *p* = 0.000	−0.013	0.007

Italian *N* = 201; Hungarian *N* = 192.

T-test for independent samples showed that the two groups have different perception of university mattering (Awareness: *t* = 2.35, *p* < 0.05; Importance: *t* = 2.05, p < 0.05; Reliance: *t* = 6.80, *p* < 0.001) and for the total score (*t* = 3.98, *p* < 0.001). In general, Hungarian participants (Awareness: *M* = 2.98, SD = 1.41; Importance: *M* = 4.66, SD = 1.37; Reliance: *M* = 3.50, SD = 1.48; Total score: *M* = 3.75, SD = 1.16) reported a higher perception of university mattering then Italian students (Awareness: *M* = 2.67, SD = 1.17; Importance: *M* = 4.40, SD = 1.12; Reliance: *M* = 2.56, SD = 1.24; Total score: *M* = 3.34, SD = 0.88).

## 5 Discussion

As described in the introduction, students’ university experience is commonly characterized not only by opportunities, but also by challenges, which can affect negatively students’ well-being (Auerbach et al., 2018; Brett et al., 2023). Some research has identified perception of mattering as a factor able to buffer these risks by enhancing university students’ well-being (e.g., Flett et al., 2019). To allow the assement of perceived university mattering in different countries, the present study aimed to adapt the UM-S to the Italian and Hungarian contexts and to test its validity and reliability. The three-factor structure of the original English version was clearly replicated, indicating that the UM-S can effectively capture university mattering across its various dimensions in both the Italian and Hungarian settings. The 10 items’ factor loadings were medium to high, suggesting that they accurately reflect the underlying dimensions of mattering distinguishing between its different components. Both the overall scale and its three subscales demonstrated high internal consistency, providing strong evidence of the reliability of the Hungarian and Italian versions.

Our results provided support for the discriminant and concurrent validity of both versions of the UM-S, as the correlation between the UM-S and a measure of social support was significantly lower than the one with a measure of societal mattering in both Italy and Hungary. Evidence for the scale’s criterion-related and incremental validity also emerged: the UM-S scores were positively associated with students’ well-being and academic self-efficacy, consistent with findings from the original version (Moschella and Banyard, 2021) and previous studies (e.g., Flett et al., 2022; Paradisi et al., 2024).

Measurement invariance analysis showed that the scores from the two versions of the scale are comparable, indicating that the UM-S can be used in cross-cultural studies. Notably, Hungarian students reported a higher perception of their importance within the university community. This difference in mattering perception may be attributed to variations in the academic environments of Italy and Hungary, particularly in the differing emphasis on Student-Centered Learning Approaches (SCLA) versus Teacher-Centered Teaching Approaches (TCTA) (see Pepicelli, 2021). The European Union agenda has been promoting a shift toward a student-centered approach in higher education, focusing on skills development, socially-oriented learning, and increased student support through mentoring and counseling programs (Fedeli and Coryell, 2014). However, Italy appears somewhat reluctant to fully embrace this direction (Barbato et al., 2019). A recent national study in nursing education revealed that many classes in Italy are conducted in large lecture halls, with the majority having over 60 students (Pagnucci et al., 2015). In contrast, Hungarian higher education appears to have adopted a more balanced approach, incorporating a mix of lectures with seminars and practical courses, fostering more interactive teacher-student engagement (Fekete, 2010; Frányó and Sándor, 2024).

We should acknowledge some limitations of our study. First, we examined the validity and reliability of the Italian and Hungarian versions of the UM-S across university students as a whole. Future research could explore the scale’s invariance among different subgroups, such as by gender or ethnic background. Second, we assessed reliability solely in terms of internal consistency; future studies should collect evidence of test–retest stability. Third, self-report measures could lead to response biases or inaccuracies, so that future research could use further indicators ro assess the scale concurrent and criterion-related validity. Forth, our sample was relatively homogeneous, with most Italian participants attending the University of Florence and most Hungarian participants from Semmelweis University, limiting the generalizability of our findings. Moreover, complete measurement invariance could not be confirmed, which might impact cross-cultural comparisons. Generalizability is also limited as these scales can be used only with Italian and Hungarian speaking students. Finally, we should also acknowledge that the disparity in sample sizes of Italian and Hungarian students may have lead to biased outcomes.

Despite these limitations, our results provide strong evidence of the good psychometric properties of the Italian and Hungarian versions of the UM-S. The scale’s cross-cultural invariance allows for meaningful comparisons in international settings. Given its brevity, the UM-S can be used in interventions to assess students’ needs and evaluate the effectiveness of programs aimed at enhancing university mattering. Indeed, since in both Italy and Hungary university students face similar well-being challenges (National Youth Council Study Center, 2023), the availability of the UM-S could help universities, faculty and mental health professionals place greater focus on students’ perception of being valued and heard within their academic environments, which is essential for promoting students’ well-being (Flett et al., 2019). The scale is also valuable for research purposes, particularly in longitudinal studies, which could help establish causal links between perceived mattering, belonging, mental health, and well-being. A short scale like the UM-S is especially suitable for repeated administrations, minimizing issues of participant fatigue and disengagement.

## Data Availability

The raw data supporting the conclusions of this article will be made available by the authors, without undue reservation.
