# Supplementary material for: Assessing perception of mattering in a cross-cultural university context: validity and reliability of the Italian and Hungarian versions of the University Mattering Scale (UM-S)
Source: Front Psychol. 2024 Dec 2;15:1502661. doi: 10.3389/fpsyg.2024.1502661 (PMC11648762; doi:10.3389/fpsyg.2024.1502661)
Supplement: Supplementary file 1 [file Table_1.DOCX]

*Table 1S.* Descriptive statistics of the 10 items Italian (N = 201) and Hungarian (N = 192) version of the UM-S

|  | Italian | | | | | | Hungarian | | | | | |
| --- | --- | --- | --- | --- | --- | --- | --- | --- | --- | --- | --- | --- |
|  | Min | Max | Mean | SD | Skew | Kurtosis | Min | Max | Mean | SD | Skew | Kurtosis |
| UM-S1 | 1 | 6 | 2.62 | 1.31 | .49 | -.74 | 1 | 6 | 2.74 | 1.54 | .47 | -.95 |
| UM-S2 | 1 | 6 | 2.58 | 1.29 | .42 | -.88 | 1 | 6 | 2.96 | 1.54 | .29 | -1.02 |
| UM-S3 | 1 | 6 | 2.91 | 1.37 | .29 | -.99 | 1 | 6 | 3.38 | 1.63 | -.03 | -1.28 |
| UM-S4 | 1 | 6 | 2.59 | 1.29 | .52 | -.62 | 1 | 6 | 2.83 | 1.55 | .46 | -.89 |
| UM-S5 | 1 | 6 | 2.46 | 1.50 | .97 | -.16 | 1 | 6 | 2.30 | 1.52 | 1.05 | .01 |
| UM-S6 | 1 | 6 | 2.48 | 1.25 | .87 | .27 | 1 | 6 | 2.40 | 1.61 | .91 | -.38 |
| UM-S7 | 1 | 6 | 2.52 | 1.39 | .84 | -.21 | 1 | 6 | 2.07 | 1.51 | 1.45 | 1.06 |
| UM-S8 | 1 | 6 | 2.90 | 1.39 | .45 | -.73 | 1 | 6 | 2.58 | 1.67 | .77 | -.63 |
| UM-S9 | 1 | 6 | 2.54 | 1.28 | .54 | -.51 | 1 | 6 | 3.37 | 1.55 | -.01 | -1.09 |
| UM-S10 | 1 | 6 | 2.60 | 1.30 | .48 | -.65 | 1 | 6 | 3.63 | 1.58 | -.18 | -1.03 |

*Note.* Omega could not be computed for reliance subscale as it comprises only two items
